# Pronounced seasonal dynamics in transcription of vitamin B1 acquisition strategies diverge among Baltic Sea bacterioplankton

**DOI:** 10.1186/s40793-025-00780-9

**Published:** 2025-09-16

**Authors:** Clara Pérez-Martínez, Benjamin Pontiller, Sandra Martínez-García, Samuel Hylander, Ryan W. Paerl, Daniel Lundin, Jarone Pinhassi

**Affiliations:** 1 Centre for Ecology and Evolution in Microbial Model Systems (EEMiS), Linnaeus University, Kalmar, SE-39182 Sweden; 2 GEOMAR Helmholtz Centre for Ocean Research Kiel, Kiel, Germany; 3 Departamento de Ecoloxía e Bioloxía Animal, Universidade de Vigo, Vigo, ES-36310 Spain; 4 Department of Marine Earth and Atmospheric Sciences, North Carolina State University, Raleigh, NC 27695 USA

**Keywords:** Thiamin, Thiamine, Marine bacteria, Cyanobacteria, Metatranscriptomics, Metagenomics, Succession, Seasonality, Food web transfer

## Abstract

**Background:**

Vitamin B1 (thiamin) is essential to life; yet little is known of the regulation of its availability in marine environments or how it varies seasonally. Since microbes are the key synthesizers of the vitamin in marine environments, we here used metatranscriptomics to examine the seasonal dynamics of B1 acquisition strategies (including both uptake and synthesis pathways) in Baltic Sea bacterioplankton.

**Results:**

Elevated B1-related gene expression was observed in summer, coinciding with increased temperatures and bacterial activity and decreased nutrient availability. Different bacterial taxa exhibited distinct B1 acquisition strategies. We identified filamentous *Cyanobacteria* of the order *Nostocales* as critical to sustaining B1 production during summer, potentially compensating for limited synthesis in heterotrophic bacteria, especially for 4-amino-5-hydroxymethylpyrimidine (HMP) synthesis. Also, *Pelagibacterales* accounted for major portions of the community transcription, primarily taking up and salvaging the B1 precursor HMP during summer. This study highlights the partitioning of B1 synthesis, salvage, and uptake among microbial taxa, underscoring that transcriptional activity was more dynamic over time than changes in the genomic potential.

**Conclusions:**

We emphasize the influence of environmental conditions on microbial community dynamics and B1 cycling in general, and the potential implications of global change-induced increases in filamentous *Cyanobacteria* blooms on vitamin food web transfer in particular.

**Supplementary Information:**

The online version contains supplementary material available at 10.1186/s40793-025-00780-9.

## Background

Vitamin B1 (thiamin or thiamine; B1 herein) is a water-soluble organic molecule that acts as a coenzyme and catalyzes parts of the tricarboxylic acid (TCA) and Calvin cycle reactions, and is involved in amino acid synthesis and central carbon metabolism [1]. Despite being an essential micronutrient to all organisms, its synthesis is limited to a few groups of microbes, including certain phytoplankton and bacterial taxa [2, 3], and many microorganisms rely on exogenous uptake of this molecule [4–7]. Accordingly, vitamin B1 in the ocean creates a wide spectrum of potential food web interdependencies, which can affect biodiversity, ecosystem productivity, and element cycling.

Although B1 and its precursors are available in seawater [8–10], dissolved B1 concentrations are extremely low (pM levels) and often below the detection limit of current methods [10–12]. Field observations show that the quantity of B1 dissolved in seawater or in particulate organic matter vary over a variety of time scales [11, 13–15]. Moreover, B1 turnover rates are presumed to be high due to both abiotic [16] and microbially-mediated processes, such as the rapid transport from the B1 pool dissolved in seawater into microorganisms [17]. Imbalances between high demand and limited production of B1 can lead to episodic events of B1 deficiency that result in growth limitation for bacteria and phytoplankton, as observed both in field studies in aquatic environments [11, 12, 18] and in laboratory experiments [19, 20]. The availability of B1 is thus an important factor with the potential to shape microbial community structure and influence biotic interactions [21, 22].

Seasonality can influence both B1 and precursors availability, and hence the status of bacterial populations as providers or consumers of the vitamin [19, 23]. Nonetheless, high-resolution temporal studies of B1 acquisition patterns are still missing and could deepen the understanding of the interconnected microbial dynamics in temperate seasonal ecosystems. It is therefore essential to understand the realized status of an organism as a provider or consumer of B1 at a particular time as compared to its genetic potential, as it might change with shifts in environmental conditions [22, 24]. Gene expression analyses of single species or bacteriplankton communities (i.e. metatranscriptomics) determine the transcriptional investment that bacteria allocate to different metabolic processes, thereby providing insight into how different taxa adjust to changes in the environment [25–27]. Thus, metatranscriptomics analyses applied to time series samples across seasons can generate detailed knowledge of the temporal dynamics of bacterial populations with respect to potential B1 metabolism and production and use of B1 in relation to its vitamers.

To interpret temporal dynamics in B1 metabolism, it is important to recognize that B1 synthesis proceeds through a two-branched pathway that forms the two precursors 4-amino-5-hydroxymethylpyrimidine (HMP) and 4-methyl-5-(2- phosphoethyl)-thiazole (THZ). The two are then combined to form thiamin monophosphate (TMP), which gets phosphorylated to the active form of the vitamin, thiamin pyrophosphate (TDP) [2]. Alternatively, intact B1 or precursors can be taken up by transporters or salvaged from degradation products to re-enter the synthesis pathway. Differences in B1 synthesis and acquisition strategies result in bacteria acting as synthesizers or complete or partial auxotrophs – i.e. pyrimidine (HMP) auxotrophs, thiazole (THZ) auxotrophs, or pyrimidine and thiazole (dual) auxotrophs [28–31]. Since microbial taxa differ in their strategy to obtain B1, the type of auxotrophy - i.e., which specific molecule in the B1 metabolic pathway an organism can synthesize or is auxotrophic for - can have important ecological consequences. Understanding how organisms are connected through precursor availability and auxotrophy could shed light on the structuring of microbial communities and their influence on processes like organic matter degradation and nutrient cycling.

To evaluate the role of microorganisms in the provision of B1 in aquatic ecosystems, it is essential to distinguish between B1 producers and consumers. Taxa known as B1 prototrophs, or *de novo* synthesizers, are taxa that have the potential to be self-sufficient in producing the vitamin. In contrast, B1 auxotrophs lack the capability to synthesize the full B1 molecule or parts thereof, so they meet their B1 demands by taking up the entire vitamin or vitamers (vitamin related compounds, including precursors) from the surrounding water [4]. They thereby avoid parts of the otherwise metabolically costly process of full B1 biosynthesis and instead rely on membrane transporters to assimilate B1 or vitamers from seawater, or by salvaging B1 precursors from degradation products [2, 24, 32]. It has been suggested that B1 prototrophs could also benefit from taking up B1 or precursors, acting as partial or entire B1 consumers [33]. B1 auxotrophy is widespread in eukaryotic phytoplankton [1, 34–36] and has also been studied in isolates of heterotrophic bacteria [3, 13, 19, 28, 37]. Recent analyses of metagenomes from the Baltic Sea, as well as freshwater and seawater systems, showed that bacterial B1 auxotrophy is much more widespread and temporally dynamic than previously recognized, with pronounced seasonal shifts in the relative abundance of genes encoding different B1 acquisition strategies [19]. These observations underline the importance of deepening our knowledge about bacterial B1 metabolism in the natural environment.

Recent studies have noted a seasonal decoupling between B1 availability in phyto- and zooplankton and its food web transfer efficiency. For instance, although filamentous *Cyanobacteria* contain high levels of B1, periods of cyanobacterial dominance during the summer correspond to the lowest B1 transfer efficiency to copepods [15, 38]. Multicellular organisms at higher trophic levels, such as zooplankton or fish, are not able to synthesize B1, and B1 deficiency in diverse macrofauna associated with aquatic environments is a worldwide concern [39–45]. This has been observed in the Baltic Sea, where Thiamin Deficiency Syndrome (named M74) leads to extensive reproduction failure of salmonid fish and waterfowl [39, 41]. Therefore, in order to understand the influence of B1 on aquatic ecosystem dynamics, it is fundamental to comprehend the regulation of B1 synthesis and acquisition mechanisms by bacteria and phytoplankton at the base of the food web.

In this study we aimed to determine the seasonal variation in transcription by bacterioplankton of genes involved in B1 metabolism. We performed metagenomic and metatranscriptomic analyses of field samples collected over two years (2016–2017, 33 time-points) in surface waters from the Linnaeus Microbial Observatory (LMO) station in the Baltic Sea Proper. Quantification of the relative transcription levels of key genes involved in B1 synthesis, uptake, and salvage was combined with identification of the temporal succession of the main taxa accounting for the transcriptional activity of B1-related metabolisms. Moreover, we identified the expressed strategies regarding B1 acquisition compared to the metagenomic potential. We reasoned that, ultimately, these analyses have the potential to provide cues on the causes and regulation of B1 sufficiency and deficiency in aquatic food webs.

## Materials and methods

### Study site and sample collection

**Fig. 1:**
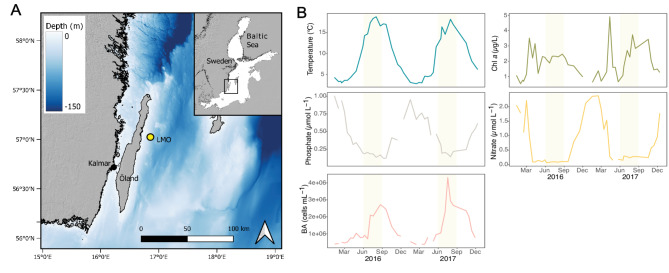
Location of the Linnaeus Microbial Observatory (LMO) and temporal dynamics in environmental and microbial variables. (**A**) Bathymetric map of the Baltic Sea Proper with the LMO station (yellow symbol) 11 km off the coast of the island Öland, Sweden. Map was drawn using data from EMODnet Digital Bathymetry [50]. (**B**) Values for temperature, chlorophyll *a* (Chl *a*), phosphate (PO_4_^3−^) and nitrate (NO_3_^−^) concentrations and bacterial abundance (BA) at LMO for the period 2016–2017. Yellow shading indicates summer periods

Seawater samples were collected biweekly from the Baltic Sea Proper at the Linnaeus Microbial Observatory off-shore station (LMO; 56° 55.8540’ N, 17° 3.6420’ E; Fig. 1A) at 2 m depth from March 2016 to December 2017 using a Ruttner sampler. Further details on the sampling station can be found in work from Lindh [46], Legrand [47] and Bunse [48]. A comprehensive summary of abiotic and biotic conditions as well as seasonal dynamics at this sampling station is available in Fridolfsson et al. [49]. Sampling frequency was approximately twice monthly from March 2016 to December 2017. Temperature and salinity were measured on-site. Additional temperature profiles from the 40 m deep water column were recorded with a CTD probe (AAQ 1186-H, Alec Electronics, Japan). Dissolved inorganic nutrient concentrations (nitrate and phosphate) and biotic parameters (Chl *a* and bacterial abundance) were analyzed as described in the work from Bunse et al. [48]. For collection of DNA, around 4 L of seawater was filtered through a Sterivex filter (GP, 0.22-µm-pore-size) approximately 1.5 h after sampling. For RNA, samples were filtered in an onshore laboratory (2–5 L, approximately 30 min after collection) through two Sterivex filters, fixed with 1.8 mL RNAlater (Qiagen, Hilden, Germany), and transported with dry ice to the laboratory. Both DNA and RNA samples were stored at -80 °C until extraction.

### Nucleic acid extractions and sequencing

DNA extraction from Sterivex filters was done by a phenol-chloroform protocol, including a 30 min lysozyme digestion step at 37 °C and a proteinase K digestion overnight at 55 °C, as detailed in Boström et al. [51]. For each sample, 2–10 ng of DNA was prepared with the Rubicon ThruPlex kit (Rubicon Genomics, Ann Arbor, Michigan, USA) according to the manufacturer’s instructions. Cleaning was done with MyOneTM superparamagnetic beads (Invitrogen, Carlsbad, CA, USA). DNA libraries were sequenced at SciLifeLab Stockholm, Sweden, on a HiSeq 2500 (Illumina Inc., San Diego, CA, USA) using a 2 × 100 bp paired-end reads setup.

For RNA extraction, samples were thawed on ice and total RNA was extracted using RNeasy Mini Kit (Qiagen) following a protocol adapted from Poretsky et al. [52] as described in Pontiller et al. [53]. In brief, DNA was removed with TURBO DNA-free Kit (ThermoFisher Scientific), and ribosomal RNA was depleted using RiboMinus Transcriptome Isolation Kit and RiboMinus Concentration Module (Thermo Fisher Scientific) after which linear amplification was done. RNA yield was measured using Nanodrop 2000 (Thermo Fisher Scientific). RNA concentration was quantified with Qubit 2.0 (Invitrogen). RNA samples were kept at -80 °C until sequencing at the Swedish National Genome Infrastructure, SciLifeLab, Stockholm. Library preparation was done with Illumina TruSeq Stranded mRNA, with no depletion or selection. The RNA samples from 2016 were sequenced on a HiSeq 2500 V4 (Illumina), PE 2 × 125 bp in 4 lanes. For the 2017 samples, sequencing was done using Multiplex in 1 lane on a NovaSeq 6000 S1 (Illumina), PE 2 × 150 bp.

### Bioinformatics

Metatranscriptomics (RNA) and metagenomics (DNA) sequencing datasets were analyzed following separate workflows. Illumina adapters were removed from RNA sequences using Cutadapt (v2.3) [54], and quality-trimmed using Sickle (v1.33) [55]. The reads were subsequently mapped to an inhouse rRNA database to remove prokaryotic ribosomal and mitochondrial RNA using Bowtie2 (v2.3.4.3) [56]. Illumina adapters in the DNA data were removed and reads quality checked with Trimgalore (v.0.6.1) [57]. After quality check, reads of both types were mapped to the BARM database [58], an interface to meta-omic data from the Baltic Sea, using Bowtie2 (v2.3.4.3) [56]. Open Reading Frames (ORFs) from the BARM database were determined using Prodigal (v2.6.3) [59] and taxonomically annotated by alignment to the NCBI RefSeq protein database with Diamond (v0.9.29) [60] and subsequent processing with MEGAN (v6.14.3) [61]. Quantification of protein-coding genes expressed in LMO samples was performed with Featurecounts from the Subread package (v2.0.0) [62]. The expression of individual genes was quantified as transcripts per million (tpm), i.e., gene length-adjusted relative abundance of individual genes in the transcriptomics sequencing in relation to the number of total prokaryotic reads remaining after featurecounts. Analogously, the relative abundance of individual genes in the metagenomes was calculated as counts per million (cpm), i.e., gene length-adjusted relative abundance of individual genes in relation to the number of total prokaryotic reads in the metagenome sequencing remaining after featurecounts. Information on sequencing depth and proportion of rRNA per sample is available in Supplementary Table S1.

**Fig. 2:**
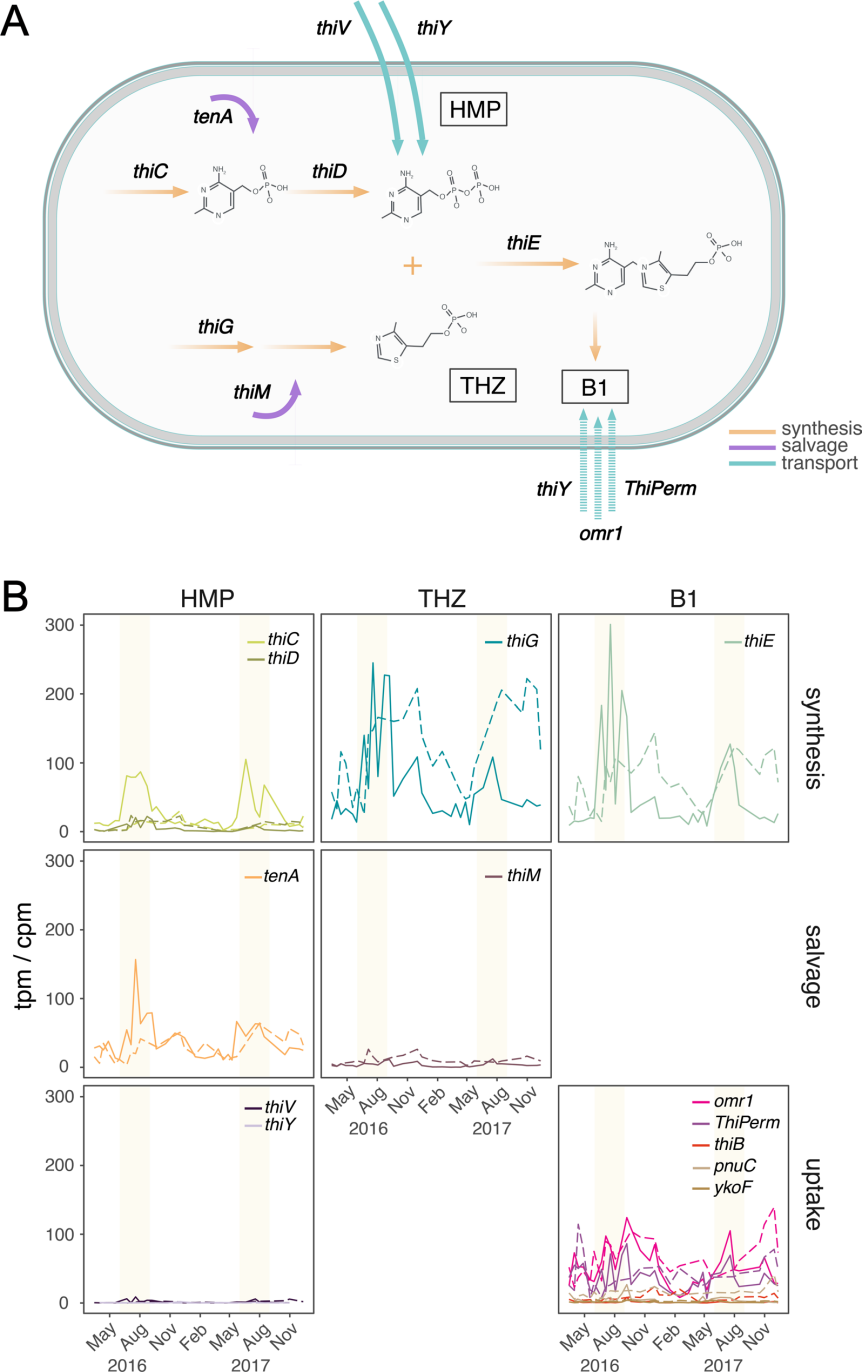
Vitamin B1 metabolic pathways and LMO metatranscriptomics. (**A**) Schematic representation of B1 synthesis (orange), salvage (purple), and transport (blue) mechanisms in bacteria. Dashed arrows (used for omr1, ThiPerm) are used for transporters whose specificity are unclear. (**B**) Transcript and gene abundance in transcripts per million (tpm) and counts per million (cpm), respectively, of key genes involved in the different branches of B1 synthesis, salvage, and uptake for the total bacterioplankton metatranscriptome (solid lines) and metagenome (dashed lines). Panels show the distribution of transcription among the branches of the B1 pathway, as well as the different acquisition mechanisms (synthesis, salvage, and transport). Transporter genes with unspecific substrate (thiamin/precursor transporters) are classified into the thiamin uptake panel. Yellow shading indicates summer periods

Functional annotation of B1 genes was done using HMM profiles described in Paerl et al. [19] (PFAM, TIGRFAM, and custom-built profiles). Marker genes for thiamin synthesis, uptake, and salvage pathways are shown in Fig. 2A. HMM profiles were aligned to LMO ORFs using hmmsearch from HMMER. Sequences scoring above trusted cutoff values were kept, ranked and the best scoring profile for each ORF was kept. High abundance ORFs in our dataset (k99_20876484 and k99_10758238) were taxonomically assigned using manual BLAST (v2.12.0) [63] against the UniProtKB/Swiss-Prot database [64] (99.48% and 99.31% identity, respectively).

### Statistical analyses and visualization

Non-Metric Dimensional Scaling (nMDS) was performed on B1 transcripts (tpm) with a Bray-Curtis distance matrix and grouped by meteorological seasons using the package *vegan* from R [65]. Seasons in the Baltic Proper were defined according to meteorological conditions in the Baltic Sea as follows: Spring: March–May, Summer: June–August, Autumn: September–November, and Winter: December–February. Community differences between seasons were examined using permutational multivariate analysis of variance (PERMANOVA) with the Bray-Curtis matrices (*vegan*). A correlation matrix between transcript abundance (in tpms) and environmental variables was calculated using Spearman correlations and represented with the *corrplot* package from R and *P*-values were calculated using the *rcorr* function from *Hmisc* package. Furthermore, pairwise correlations were computed to examine the relationship between environmental variables and gene expression across seasons.

To display expression ratios, we calculated the log2 transformed ratios between metatranscriptomics and metagenomics relative abundances for each B1 gene, taxon and time point after normalizing the number of tpm for both datasets. Positive values indicate higher relative abundance in the metatranscriptomes, while negative values represent higher relative abundance in the metagenomes. Furthermore, we assigned the genes present in the environment that were not transcribed by grouping all sequences by order and protein and selecting those that were present in the metagenomics but not metatranscriptomics datasets for every sample and referred to them as “silent” genes. This was possible since both metagenomics and metatranscriptomics datasets were mapped to the same database.

All plots were made using the *ggplot2* package in R (v4.4.0) [66].

## Results

### Seasonality in environmental and microbial variables

Our biweekly sampling at the Linnaeus Microbial Observatory (LMO) revealed seasonally recurring patterns of physicochemical conditions and biological variables, consistent with the Baltic Sea being a northerly temperate zone environment (Fig. 1). Temperatures ranged from 2.8 °C to 18.8 °C (Fig. 1B). Nitrate concentrations were high during the period October-March (reaching up to 2.38 µM) and rapidly decreased to values of 0.30 − 0.06 µM in April (Fig. 1B). Phosphate concentrations were also high in winter (up to 1.01 µM) and decreased slowly during spring, reaching ~ 0.12 µM in July and August (Fig. 1B). Chlorophyll-*a* (Chl *a*) concentrations displayed distinct spring and summer peaks in both years (Fig. 1B). In 2016, the spring bloom occurred from March to April, with concentrations peaking at 3.5 µg Chl *a* L^− 1^ and gradually declining to 1.2 µg L^− 1^. From May to September, Chl *a* concentration varied around 2 µg L^− 1^ before gradually decreasing to winter levels of 0.6 µg L^− 1^. In 2017 a more pronounced spring bloom took place, reaching up to 4.9 µg Chl *a* L^− 1^ in April, and decreasing to 0.6 µg L^− 1^ in May. The summer bloom, lasting from July and well into autumn, reached 3.7 µg Chl *a* L^− 1^ and declined after October. Bacterial abundance was at its lowest during late winter (~ 3.8 × 10^5^ cells mL^− 1^) in both years but showed a rapid increase to over 2.0 × 10^6^ cells mL^− 1^ in late June and remained high until October (Fig. 1B).

### Seasonality in bacterioplankton community vitamin B1 expression and gene abundance

Overall, the transcriptional investment in B1 metabolism averaged 0.08% of the total bacterioplankton community gene expression and was divided between synthesis (52% of total B1 transcription), salvage (15%), and uptake (33%) (Table 1). All three pathways showed pronounced temporal variability, with distinct summer peaks and low expression during winter for most of the B1-related genes (Fig. 2B). For synthesis marker genes, the transcription levels of THZ synthesis *thiG* (thiazole synthase) and B1 synthesis *thiE* (thiamin-phosphate synthase) were coupled, reaching values of 245 and 301 tpm, respectively, in the summer months, while the transcription of the HMP synthesis gene *thiC* (phosphomethylpyrimidine synthase) reached maximum levels of 105 tpm. Expression of *thiD* (hydroxymethylpyrimidine/phosphomethylpyrimidine kinase) was lower, only reaching 22 tpm (Fig. 2B). The transcription of the HMP salvage gene *tenA* (aminopyrimidine aminohydrolase) peaked in summer (156 tpm), showing a similar pattern to that of the *thiG* and *thiE* genes, while THZ salvage (hydroxyethylthiazole kinase, *thiM*) expression was consistently low (up to 12 tpm). Transcripts for B1/precursor uptake were more stable during the year, with a drop in winter. The *omr1* gene reached transcription levels up to 124 tpm, while the transporter *ThiPerm* reached 86 tpm. The other transporters included in the analysis were only detected at low transcription levels (Fig. 2B). Notably, the presence of B1 genes in the metagenome differed from gene expression patterns. For synthesis genes, *thiG* and *thiE* showed the highest metagenomic relative abundances, reaching 222 and 144 cpm, respectively, with peaks in August and October-December. The HMP synthesis genes *thiC* and *thiD* had lower maximum values (30 and 24 cpm, respectively). The HMP salvage gene *tenA* peaked in the same months as *thiG* and *thiE*, reaching 64 cpm. Among uptake genes, *omr1* was more consistently detected, with a maximum of 139 cpm and peaks from September to December, while *ThiPerm* reached 114 cpm, peaking in April and October–December.

**Table 1:** Taxonomic distribution of total community transcription versus transcriptional investment (mean values) in B1 acquisition mechanisms at LMO, using the sum of B1 synthesis, salvage or uptake genes

	Proportion of total metaT (%)	Proportion of B1 metaT (%)	Ratio of B1 to total metaT	log2 ratio (B1:total metaT)	Synthesis % (relative to total B1 metaT)	Salvage % (relative to total B1 metaT)	Uptake % (relative to total B1 metaT)
*Actinobacteria*	3.82	3.21	0.84	-0.25	1.83	0.21	1.17
*Alphaproteobacteria*	24.71	41.88	1.70	0.76	19.38	9.61	12.89
*Bacteroidetes*	5.50	3.89	0.71	-0.50	0.80	0.69	2.40
*Betaproteobacteria*	3.15	3.45	1.09	0.13	1.41	1.65	0.39
*Cyanobacteria*	36.68	18.77	0.51	-0.97	17.90	0.15	0.72
*Gammaproteobacteria*	11.68	10.69	0.92	-0.13	2.22	1.35	7.12
Other *Proteobacteria*	4.51	8.20	1.82	0.86	2.46	0.88	4.85
*Planctomycetes*	1.00	0.64	0.64	-0.64	0.48	0.13	0.02
*Verrucomicrobia*	1.68	3.42	2.04	1.03	1.58	0.21	1.64
Other	7.27	5.85	0.80	-0.31	3.47	0.65	1.73
Total investment of the community (%)	100	100	*-*	*-*	51.55	15.52	32.93

**Fig. 3:**
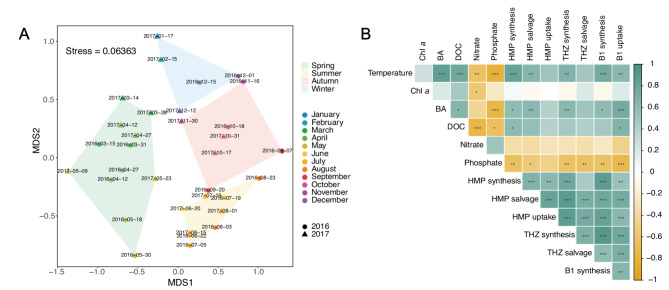
Clustering of the B1 metatranscriptomics samples and correlation with environmental variables. (**A**) Non-metric dimensional scaling (nMDS) plot showing the seasonal differences in all B1-related transcripts. Samples are represented by month (color) and year (shape) and grouped by season. (**B**) Correlation matrix between environmental variables (temperature, Chlorophyll *a* [Chl *a*], bacterial abundance [BA], dissolved organic carbon [DOC], nitrate, and phosphate) and gene expression of different branches and acquisition strategies of B1 based on Spearman correlations. Color intensity indicates correlation strength. The legend shows Spearman correlation coefficients. Significance is indicated by ***, **, * for *p*-values 0.001, 0.01 and 0.05

Non-metric multidimensional scaling (NMDS) revealed a significant grouping of B1 transcripts from the two years into seasonal clusters (Fig. 3A, NMDS stress = 0.06). PERMANOVA analysis indicated that grouping the transcripts by meteorological season explained a significant proportion of the variation in transcript composition (R²=0.3, *p* < 0.001). Spearman rank-based correlation analysis generally showed significant positive correlations between the relative expression of B1 genes and temperature and negative correlations with inorganic nutrients (phosphate) (R^2^-values in the range 0.31–0.59) (Fig. 3B). Heterotrophic bacterial abundance was positively correlated with the expression of B1 genes, whereas changes in Chl *a* did not correlate with bacterial B1 expression. Furthermore, there was a positive correlation between the expression of genes in the different branches of B1 synthesis, uptake, and salvage (Fig. 3B). In particular, the synthesis of B1 was strongly positively correlated to the synthesis of both precursors (R^2^ = 0.66, *p* < 0.001for HMP; R^2^ = 0.82, *p* < 0.001 for THZ). Visualization of pairwise correlations separated per season showed that the relationships between environmental conditions and the different acquisition mechanisms and pathways of B1 metabolism changed throughout the year (Supplementary Figure S1). For example, the relationship between Chl *a* concentrations and transcripts in all the B1 acquisition mechanisms and branches, except for HMP synthesis, varied from a negative correlation in spring to positive in summer.

### Taxonomic distribution of B1 genes

To characterize the temporal variation of B1-related transcripts, we analyzed the dynamics in key taxa responsible for the transcriptional changes observed. *Alphaproteobacteria* were highly active, accounting for up to 42% of transcriptional investment in B1 metabolism, which was approximately double their expected contribution based on their relative proportion in the total community’s transcription. This was reflected in a B1 to total community transcription ratio of 2.3 (Table 1). Around 19% of B1 metabolism transcripts were attributed to *Cyanobacteria*, which dominated synthesis transcription in most samples, although their investment in B1 transcription was lower than expected from their total transcription (B1/total transcription ratio = 0.7). Following that, *Gammaproteobacteria* accounted for 11% of B1 transcription, and *Betaproteobacteria*, *Bacteroidetes* and *Actinobacteria* each contributed with around 3–4% of B1 transcripts. *Verrucomicrobia* had a remarkably higher investment in B1 (3.4%) compared to their total community transcription (B1/total transcription ratio = 2.7). For the remaining taxa, B1 expression was proportional to their total transcription (Table 1).

**Fig. 4:**
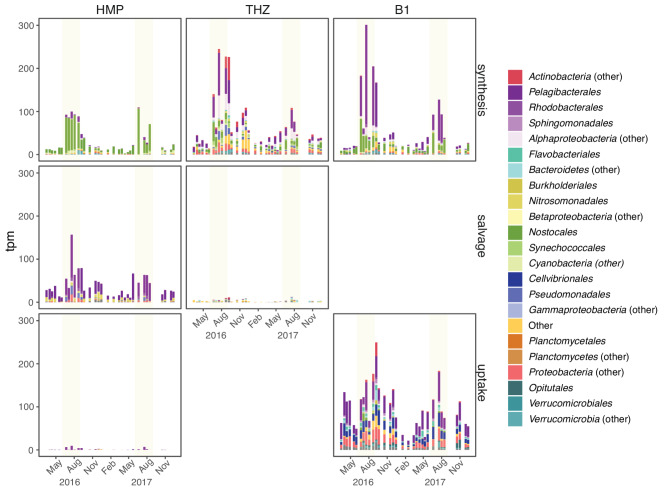
Relative transcript abundances for the top taxa (orders) expressing B1 genes at LMO through the two-year period. The panels show the distribution of transcription among the different branches of the B1 synthesis pathway, as well as the different acquisition mechanisms (synthesis, salvage, and transport). Transporter genes with unspecific substrate (thiamin/precursor transporters) are classified into the thiamin uptake panel. Yellow shading indicates summer periods

Taxonomy-resolved transcription of B1 synthesis, uptake, and salvage pathways provided further detail on the dynamics of B1 metabolism (Fig. 4). *Cyanobacteria* largely dominated the HMP synthesis expression throughout the year (Fig. 4). *Alphaproteobacteria* dominated the expression of B1 synthesis, salvage, and uptake pathways in the heterotrophic portion of the bacterioplankton community (Fig. 4). *Pelagibacterales* (*Alphaproteobacteria*) dominated the THZ and B1 synthesis branches (the latter with a contribution of up to 27% by *Cyanobacteria*). *Pelagibacterales* also dominated HMP salvage and uptake. Finally, transcription of B1 and vitamer uptake genes was carried out by a large diversity of bacterial taxa and presented higher relative transcription values during spring and autumn than the other acquisition mechanisms (Fig. 4).

**Fig. 5:**
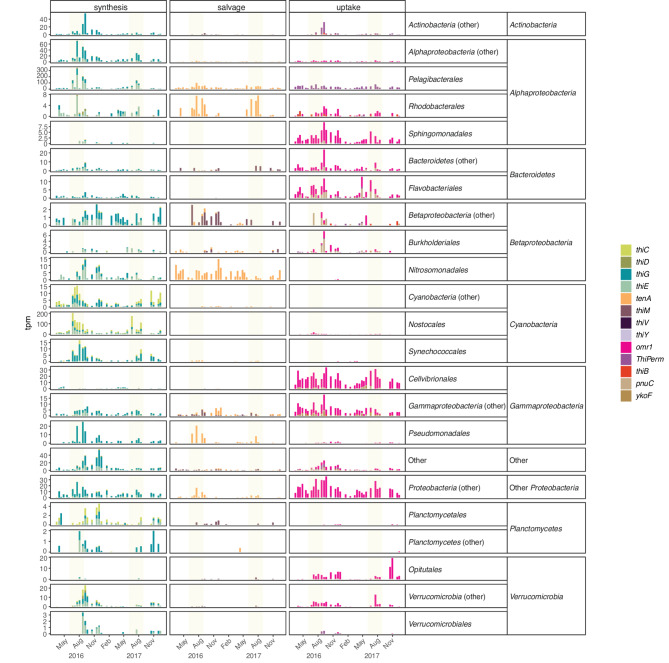
Taxonomic distribution of B1 transcripts (tpm) across the main taxa contributing to B1 gene expression. Yellow shading indicates summer periods

Detailed analysis of the temporal dynamics of the key taxa contributing to B1 metabolism in the community showed that *Alphaproteobacteria* did not express *thiC*, indicating they did not synthesize HMP. *Pelagibacterales* largely dominated the alphaproteobacterial transcription, with elevated transcription of the THZ synthesis genes *thiG* (up to 98 tpm) and *thiE* (up to 230 tpm), particularly during the summer months (Fig. 5). Coupled with that was the expression of the *tenA* gene (up to 100 tpm) for HMP salvage. Regarding the transcription of transporters by *Pelagibacterales*, we observed consistently high levels of ThiPerm gene expression throughout the year (reaching 66 tpm). Expression values for the HMP transporter *thiV* were low, reaching up to 9 tpm. Notably, different *Pelagibacterales* strains expressed a distinct set of B1-related genes (Supplementary Fig. 2): *Candidatus* Pelagibacter ubique expressed the synthesis genes *thiG* and *thiE*, along with the transporter *ThiPerm*; Alpha proteobacterium HIMB114 expressed *thiG*, *thiE*, and the salvage gene *tenA*; while Alpha proteobacterium HIMB59 expressed only the transporter genes *thiY* and *thiB*. A similar pattern to *Pelagibacterales*, but at lower transcription levels, was observed in *Rhodobacterales*, which expressed *thiG* and *thiE* genes for THZ and B1 synthesis but lacked *thiC* expression. In contrast, *Sphingomonadales* mainly took up B1, based on the expression of the *omr1* transporter.

*Cyanobacteria* were virtually all net B1 synthesizers, with expression peaks in summer (Fig. 5). *Nostocales* dominated cyanobacterial B1 synthesis transcription and had the highest relative expression of *thiC* for the entire bacterial community (reaching 105 tpm) as well as elevated levels of *thiG* (up to 44 tpm) and *thiE* (up to 77 tpm). They also expressed the B1 transporter gene *omr1* (up to 18 tpm). At lower levels, *Synechococcales* were also expressing the HMP and THZ synthesis genes *thiC* and *thiG* (Fig. 5).

*Gammaproteobacteria* primarily expressed genes for B1 uptake; but while none of them expressed the HMP synthesis marker *thiC*, they exhibited lower summer peaks of transcription of *thiG* and *thiE* synthesis genes (Fig. 5). The dominating group to total B1 expression throughout most of the study period was *Cellvibrionales*, which expressed fair amounts of the *omr1* transporter (up to 33 tpm). Contrastingly, the *Pseudomonadales* group expressed the *thiG* gene for THZ synthesis (up to 25 tpm) and salvaged HMP using the *tenA* gene (up to 21 tpm).

Among other taxa, we found a wide variety of B1 acquisition strategies. In general, *Betaproteobacteria* expressed genes for THZ and B1 synthesis and HMP salvage. The most active group within *Betaproteobacteria*, *Nitrosomonadales*, did not express *thiC* for HMP synthesis but expressed the *thiG* and *thiE* synthesis genes and the HMP salvaging gene *tenA*. *Bacteroidetes* expression was dominated by the uptake genes *omr1* and *pnuC*. They did not exhibit HMP synthesis expression but expressed THZ and B1 synthesis genes. *Actinobacteria*, in turn, predominantly relied on the *ThiPerm* transporter for uptake. They did not synthesize HMP, but actively synthesized THZ using the *thiG* gene (up to 54 tpm). *Verrucomicrobia* exhibited both net synthesis of all B1 branches and uncoupled uptake using the *omr1* transporter (up to 20 tpm), a strategy used by *Opitutales* within this taxon. Finally, *Planctomycetes* were generally synthesizers of both B1 and precursors.

### Active bacterioplankton vitamin B1 transcription

**Fig. 6:**
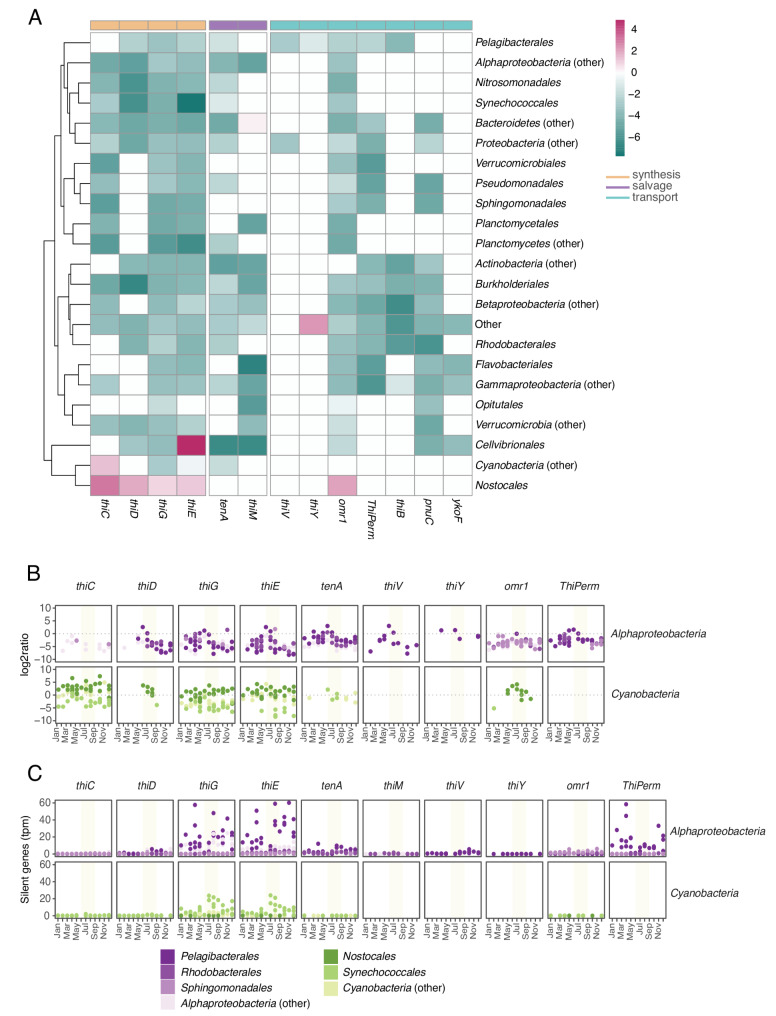
Temporal dynamics in relative expression levels of B1 genes in metatranscriptoes (metaT) in relation to the relative abundance of the genes in the metagenomes (metaG). (**A**) Heatmap showing the log2 transformed ratios between metaT and metaG relative abundances for each B1 gene and taxon. Pink colors are showing higher metaT than metaG, while green shows higher relative abundance of the gene than transcription. (**B**) Plot showing log2 transformed ratios between metatT and metaG relative abundances for each B1 gene, taxon, and time point. Positive values indicate higher relative abundance in the metaT, while negative values represent higher relative abundance in the metaG. Full representation of the bacterial community in Supplementary Figure S3. (**C**) Abundances of silent genes (genes in the metagenomic dataset and not present in the metaT), for each of main taxa contributing to B1 expression at LMO across the year. Each point represents a sample. Full representation of the bacterial community in Supplementary Figure S4. Yellow shading in panels B and C indicates summer periods

We next analyzed the role of bacterioplankton as potential producers or consumers of B1 in the natural environment in relation to their metagenomically encoded potential. For that, we calculated expression ratios, the ratio between the contribution of a gene to the metatranscriptome and its contribution to the metagenome (tpm metatranscriptome / cpm metagenome) - where a ratio of 1 implies a one-to-one relation between the relative abundance in the metaT and metaG data (Fig. 6). This analysis showed that, for a majority of the studied genes, the relative contribution to the metatranscriptome was lower than the corresponding contribution to the metagenome. However, the *Nostocales* stood out by showing ratios higher than one for all synthesis genes (Fig. 6A).

The expression ratios of different genes exhibited varied temporal dynamics (Fig. 6B, Supplementary Figure S3), indicating seasonal changes in the performance of B1 acquisition strategies. For example, changes in *Pelagibacterales* gene expression ratios occurred intermittently throughout the year, with fluctuations in expression levels observed across different genes and time periods. Note particularly that the expression ratio for genes encoding partial B1 synthesis (*thiG*, *thiE*), HMP salvage (*tenA*) and uptake (*ThiPerm*) increased during the summer for this group. In contrast, *Nostocales* showed persistently elevated metaT: metaG ratios over the entire study period with relatively little variation over time (Fig. 6B).

Furthermore, as an extreme case of underrepresentation in the metatranscriptomes, we identified what we denote “silent genes”, i.e. genes that were found in the metagenome but not detected in the metatranscriptome (Fig. 6C, Supplementary Figure S4). Many of the *thiG*, *thiE*, and *ThiPerm* genes in *Pelagibacterales* genomes were “silent” during most of the year. Similarly, *Synechococcales* presented high numbers of silent *thiG* and *thiE* genes from July to December. Overall, these findings highlight the diverse and dynamic expression patterns of different taxa, and their genes involved in B1 metabolism.

## Discussion

The overall changes in relative expression patterns we observed suggest that seasonality, with changes in environmental conditions, is likely to influence the availability of B1 and its precursors and hence the status of bacterioplankton populations as providers or consumers of the vitamin. The relationships between B1-related gene expression and seasonality were further noted in the positive correlation of relative expression patterns with temperature and bacterial abundance and the negative correlation with inorganic nutrients. Elevated B1 expression levels largely drove these correlations in summer when bacterioplankton were more abundant and active. These findings expand on previous knowledge that the bioavailability of B1 and/or its precursors, as well as other B vitamins, changes over time and thereby potentially plays a role in structuring the succession of bacterioplankton communities [13, 19, 23, 67].

Previous molecular studies have provided first insights into the functional roles and activity levels of microbial populations involved in B1 cycling. Accordingly, Bittner et al. [23] showed that B1 concentrations were relatively stable across seasons, suggesting a balance of supply and demand, while vitamer concentration changed markedly with season, as did transcripts related to vitamer salvage and transport [23]. Gómez-Consarnau et al. [13] investigated the gene expression dynamics of vitamin B1 synthesis (but not uptake or salvage) at a coastal location in the North Atlantic (Sapelo Island, US), attributing higher B1 concentrations in seawater to heterotrophic bacterioplankton synthesis. It is noteworthy that our observed transcription levels are similar to those of both Bittner [23] and Gómez-Consarnau [13] (< 0.01–0.04% of normalized transcripts of B1 biosynthesis genes). In a metagenomics analysis over nearly a year (33 sampling times, Mar-Nov 2012) at the same LMO station as our current study, Paerl et al. [19] showed seasonal variation in the relative abundance of bacterial B1 synthesis, salvage, and transport genes. This variation included changes ranging from weekly to seasonal scales, showing a potential for rapid dynamics in bacterial metabolism. In our current work, seasonality in the metatranscriptomes was observed for all three forms of B1 acquisition mechanisms studied - synthesis, salvage, and uptake - although with different degrees of variability. Importantly, the amplitude of seasonal variation in the metatranscriptomes was substantially larger than what was visible in the metagenomes sampled in parallel. The shifts in vitamin B1 acquisition forms resulted from dynamics in both the individual genes involved and the taxa that contributed. This implies that the observed changes in expression of B1 metabolisms over time were influenced not only by overall changes in community composition but also by changes in the relative levels of transcription of particular genes and pathways within given prokaryotic taxa. The finding that transcription strategies for B1 acquisition are substantially more dynamic over time compared to that indicated by the genomically encoded potential emphasizes the importance of disentangling both quantitative and qualitative (e.g. vitamin B1 acquisition forms) temporal changes in the metabolism of B1 and its precursors.

B1 auxotrophy is widespread among bacterioplankton, and evidence of HMP auxotrophy is consistently found in LMO metagenomes along with incidences of dual auxotrophy (i.e. requirement for both HMP and THZ) and intact B1 auxotrophy [19]. Our observations on the bacterioplankton community transcription also showed that there was a coupling between HMP salvage, the synthesis of THZ, and the merging of the two precursors. Moreover, eukaryotic phytoplankton (primarily diatoms and dinoflagelates) that form the pronounced Baltic Sea spring blooms could play a role in producing and/or consuming B1, and thereby influence the availability of B1 to bacteria, as indicated by the decoupling between Chl *a* concentrations and relative B1 expression. Several eukaryotic phytoplankton species can synthesize B1 [68] and, for example, it has been proposed that a periodic increase in diatom abundance can foster the bioavailability of THZ [30]. We thus find it likely that the availability of B1 is influenced by phytoplankton species composition and abundance [38, 69]. Further studies should therefore delve deeper into understanding the role of eukaryotic phytoplankton on the B1 metabolism of the microbial community as a whole, which would likely provide a broader ecological context. Yet, even without detail on the potential role of phytoplankton populations as providers of B1 or its vitamers, our results highlight the importance of different bacterial B1 acquisition strategies and the temporal development of bacterial taxa as producers or consumers of the vitamin.

Several major bacterioplankton taxa have been described as HMP auxotrophs, including the abundant *Pelagibacterales* group, as they lack the HMP synthesis gene *thiC* [19, 28]. Moreover, specific *Pelagibacterales* strains can also be intact B1 consumers [28]. The high contribution of *Pelagibacterales* to the community-wide relative expression of B1 metabolism, in our study, highlighted the role of this taxon in B1 cycling in the Baltic Sea. We provided new insights into *Pelagibacterales* potential requirements for exogenous B1 that were not previously described, as ThiPerm expression was fairly stable over time (relative to the total community). Furthermore, the high *tenA* transcription levels indicated the importance of HMP salvage. Lastly, the great variation in the contribution of *Pelagibacterales* to the bulk THZ and thiamin synthesis throughout the dataset provided novel insights into the ecological role of this taxon in the seasonal partitioning of microbial B1 metabolism. For instance, the abundant and transcriptionally active *Pelagibacterales* mostly relied on the uptake of B1 or its precursors. The expression patterns of the different *Pelagibacterales* strains described in our study (Supplementary Figure S2) corresponded to the ones described in the work by Carini et al. [28]. Moreover, the expression of genes for HMP uptake and salvage peaked in summer when filamentous *Cyanobacteria* expressed high levels of genes for HMP synthesis, as compared to the rest of the year when they favored the uptake of intact B1. This strategy allows *Pelagibacter* cells to avoid the costs of full vitamin B1 synthesis [70].

Similar to *Pelagibacterales*, the relative expression of *Rhodobacterales* was geared towards THZ and B1 synthesis and HMP uptake, although a portion of *Rhodobacterales* expressed genes for direct uptake of intact B1. Thus, although described as a dual auxotroph [19]. *Rhodobacterales* seem to encompass populations that vary in auxotrophy types. In contrast to other *Alphaproteobacteria*, our findings of elevated relative expression of *omr1* in *Sphingomonadales*, supported previous suggestions that they are intact B1 auxotrophs [19]. Regarding other major taxa, relative expression data presented here suggested that most *Gammaproteobacteria* - and particularly *Cellvibrionales* - behaved as thiamin consumers, in line with reports that *Cellvibrionales* are obligate B1 auxotrophs since they lack the *thiE* gene [19]. An interesting observation was that the picocyanobacterial *Synechococcales* displayed gene expression for the complete metabolic pathway to synthesize B1. This was similar to the filamentous *Cyanobacteria* (see below), although the transcription of HMP synthesis gene *thiC* was relatively lower; an interpretation of this curious finding awaits future comparative work on B1 biosynthesis transcription patterns in different organisms. In summary, the distribution of transcriptional investment in B1 synthesis and utilization pathways among bacterial taxa underscores the compartmentalization of these metabolic processes and emphasizes the ecological significance of individual taxa in B1 cycling dynamics.

In their description of the seasonal changes in the reliance of bacterioplankton upon B1 or precursors, based on relative gene abundances in metagenomes, Paerl et al. [19] observed a substantial deficiency in the potential capacity to synthesize HMP in microbial communities from the Baltic Sea. Yet, it should be noted that filamentous *Cyanobacteria* (*Nostocales*) were largely excluded in that study due to the prefiltration applied. Curiously, Paerl [19] observed that the prevalence of heterotrophic HMP auxotrophs was positively correlated to the biomass of filamentous *Cyanobacteria* (measured by microscopy). Our current analysis on samples without prefiltration (thus including *Nostocales*) confirm that the relative abundance of HMP synthesis genes (*thiC*/*D*) in the microbial community was about 5–10 times lower than that of synthesis genes for HET (*thiG*) or B1 (*thiE*). This may indeed give the impression that there is a deficiency in HMP synthesis capacity in the microbial community. However, we think that the high relative expression levels of especially *Nostocales thiC* (but also *thiD*) during the productive season may compensate for the apparent deficiency of HMP synthesis genes in the microbial community. In open ocean environments, other filamentous *Cyanobacteria* like *Trichodesmium* have been noted as key contributors to HMP synthesis [71]. They also transcribed *omr1* to a notable level, so *Nostocales* may promote HMP users further by also using exogenous B1. *Nostocales* are generally rich in thiamin compared to other phytoplankton and comparisons between systems with (Baltic) or without (Skagerrak) filamentous *Cyanobacteria* demonstrate higher overall thiamin concentrations in summer phytoplankton communities dominated by filamentous *Cyanobacteria* [15, 69]. Moreover, upregulation of B1 synthesis gene expression (including *thiC* and *THI4* [in our analyses denoted *thiG*]) has been observed under stress-inducing experimental conditions in the cyanobacterial strain *Anabaena* sp. [72]. As emphasized above in general terms for community-wide metatranscriptomics analyses as carried out here, the expression levels of *Nostocales* in our data is determined by both the abundance of filamentous *Cyanobacteria* and by the relative expression level of B1 genes of that taxon. Generally, *Nostocales* reach high biomass in summer in the Baltic Sea [49], whereby the integrated result of the seasonal abundance changes and their expression profiles for B1 genes come to be important for determining their overall contribution to the community wide B1 metabolism. We think this provides an important example of how transcriptional analysis contributes to resolving *Nostocales* as a key player in HMP production during the summer and thereby provides mechanistic understanding of the relative importance of specific taxonomic groups in B1 metabolism over time.

Our findings emphasize the importance of *Nostocales* in sustaining B1 production during summer periods in the Baltic Sea. Filamentous *Cyanobacteria* are abundant in Baltic summers [47, 73, 74], and blooms of filamentous *Cyanobacteria*, often involving species that produce harmful toxins, are expected to increase in frequency and magnitude in several aquatic systems as a result of eutrophication and global warming [75–77]. In particular, the combination of increased temperatures and shifts in inorganic nutrient dynamics are likely to influence the population dynamics and B1 transcription of *Cyanobacteria* and other B1 producers - thus potentially having substantial effects on B1 production and bioavailability in the pelagic food web.

## Conclusions

In conclusion, our study unveils intricate dynamics of expressed B1 acquisition strategies employed by different bacterial taxa, showcasing the complex interplay in dictating B1 metabolism between genomic potential and community composition on the one hand and transcriptional investment on the other hand. Notably, filamentous *Cyanobacteria*, particularly *Nostocales*, emerge as key contributors to HMP production and B1 synthesis during summer blooms, potentially influencing overall B1 availability in the ecosystem. In contrast, in bacteria other than *Nostocales*, synthesis genes were generally found at lower expression levels as compared to their relative abundance in the metagenomes during summer. As uptake is generally a less costly option than synthesis, this would prevent the performance of energetically costly B1 synthesis functions when the entire B1 molecule or its precursors could be otherwise available. Collectively, these findings shed light on the importance of considering taxonomic diversity and seasonal dynamics in understanding B1 cycling and its implications for ecosystem functioning.

## Electronic supplementary material

Below is the link to the electronic supplementary material.

Supplementary Material 1

## Data Availability

Nucleotide sequence data is available at European Nucleotide Archive (ENA) under the accession number PRJEB69280 for metatranscriptomics and accession number PRJEB82694 for metagenomics.
