# Supplementary material for: Pronounced seasonal dynamics in transcription of vitamin B1 acquisition strategies diverge among Baltic Sea bacterioplankton
Source: Environ Microbiome. 2025 Sep 16;20:115. doi: 10.1186/s40793-025-00780-9 (PMC12442306; doi:10.1186/s40793-025-00780-9)
Supplement: Supplementary file 1 — Supplementary Material 1 [file 40793_2025_780_MOESM1_ESM.docx]

Supplementary Materials

| **Date** | **Raw reads in metaG** | **Raw reads in metaT** | **Proportion of rRNA in metaT (%)** |
| --- | --- | --- | --- |
| **2016-03-15** | 43494020 | 40217244 | 56.30 |
| **2016-03-31** | 40869411 | 44020457 | 56.80 |
| **2016-04-12** | 90215404 | 45367595 | 67.30 |
| **2016-04-27** | 64721580 | 44033617 | 66.60 |
| **2016-05-18** | 53451225 | 51170919 | 61.80 |
| **2016-05-30** | 46330365 | 40432990 | 62.50 |
| **2016-06-22** | 45639300 | 46164668 | 57.90 |
| **2016-07-05** | 533886773 | 45630316 | 58.80 |
| **2016-07-19** | 51636857 | 56712272 | 57.00 |
| **2016-08-03** | 41027979 | 47904800 | 57.20 |
| **2016-08-23** | NA | 53581926 | 54.20 |
| **2016-09-07** | NA | 49547145 | 48.90 |
| **2016-09-20** | 52454903 | 30567264 | 61.80 |
| **2016-10-18** | 44086172 | 58597708 | 55.80 |
| **2016-11-16** | NA | 65519011 | 50.60 |
| **2016-12-01** | 27500182 | 54256206 | 50.10 |
| **2016-12-15** | 65115613 | 56768819 | 53.00 |
| **2017-01-17** | 41622609 | 28480121 | 39.30 |
| **2017-02-15** | 46944324 | 63603505 | 44.20 |
| **2017-03-14** | NA | 65563376 | 47.90 |
| **2017-03-28** | NA | 67507214 | 54.00 |
| **2017-04-12** | 69862938 | 63980351 | 54.90 |
| **2017-04-27** | 64561353 | 72160794 | 54.20 |
| **2017-05-09** | 39958393 | 73145463 | 57.00 |
| **2017-05-23** | 33262510 | 65122068 | 56.40 |
| **2017-06-20** | NA | 67975908 | 61.60 |
| **2017-07-19** | NA | 64854477 | 62.40 |
| **2017-08-01** | 54104445 | 71128507 | 61.20 |
| **2017-08-15** | 90810277 | 68088806 | 62.10 |
| **2017-10-17** | 32473245 | 64251304 | 50.00 |
| **2017-10-31** | 30746589 | 72921796 | 49.00 |
| **2017-11-30** | 25832562 | 62548976 | 49.60 |
| **2017-12-12** | 42209124 | 70133116 | 49.40 |

**Supplementary Table S1.** Sequencing depth of metagenomics and metatranscriptomics datasets and proportion of rRNA per sample.


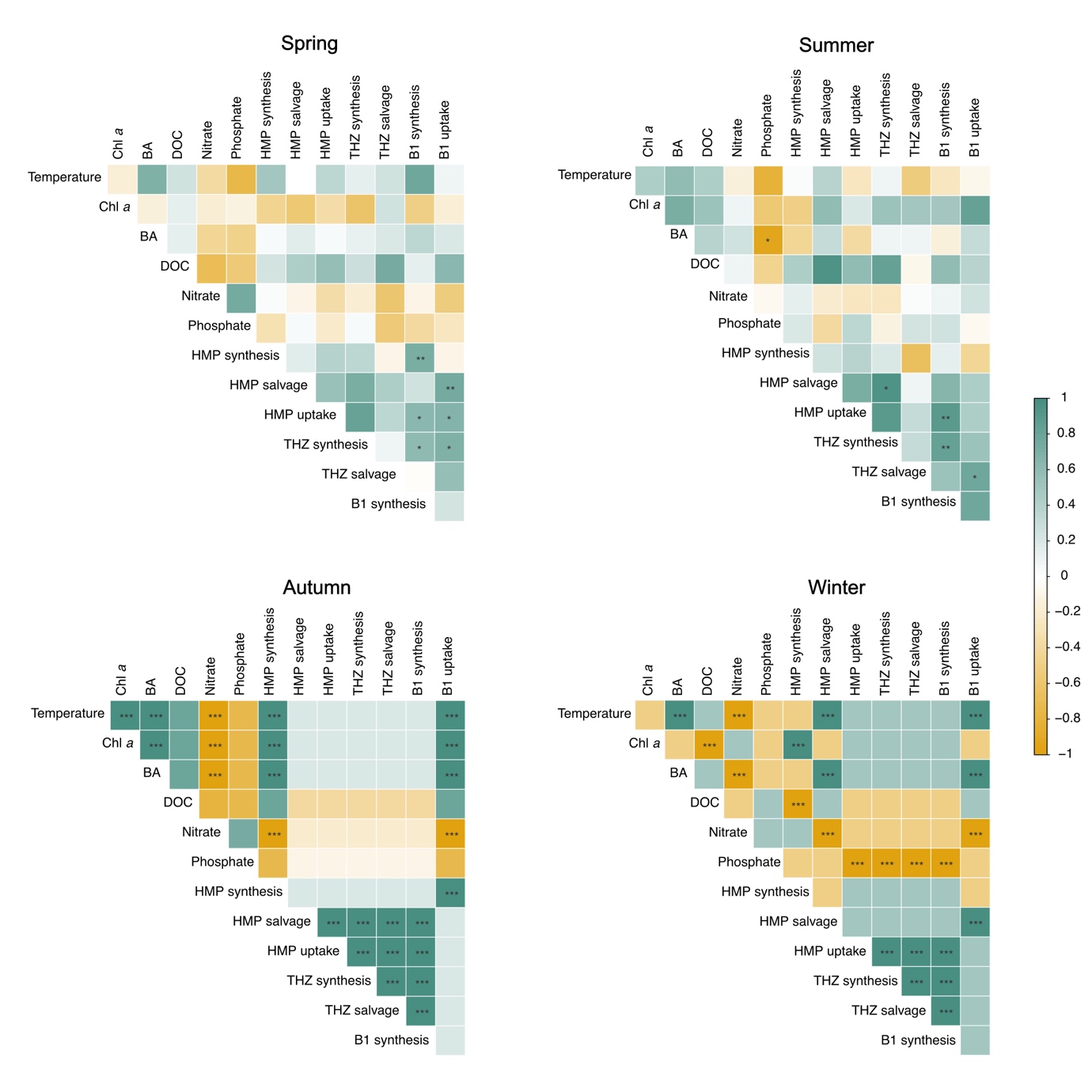


**Supplementary Figure S1.** Pair-wise correlations among seasons between environmental variables (temperature, Chlorophyll *a* [Chl *a*], bacterial abundance [BA], dissolved organic carbon [DOC], nitrate, and phosphate) and gene expression of different branches and acquisition strategies of B1 based on Spearman correlations. Color intensity indicates correlation strength. The legend shows Spearman correlation coefficients. Significance is indicated by ***, **, * for p-values 0.001, 0.01 and 0.05.


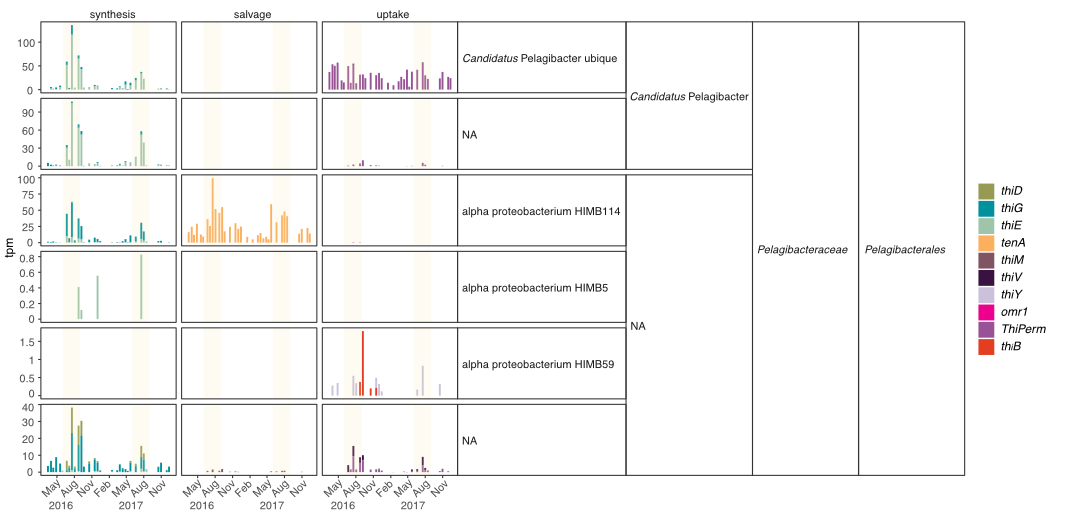


**Supplementary Figure S2.** Taxonomic distribution of B1 transcripts (tpm) across *Pelagibacterales*. Yellow shading indicates summer periods.


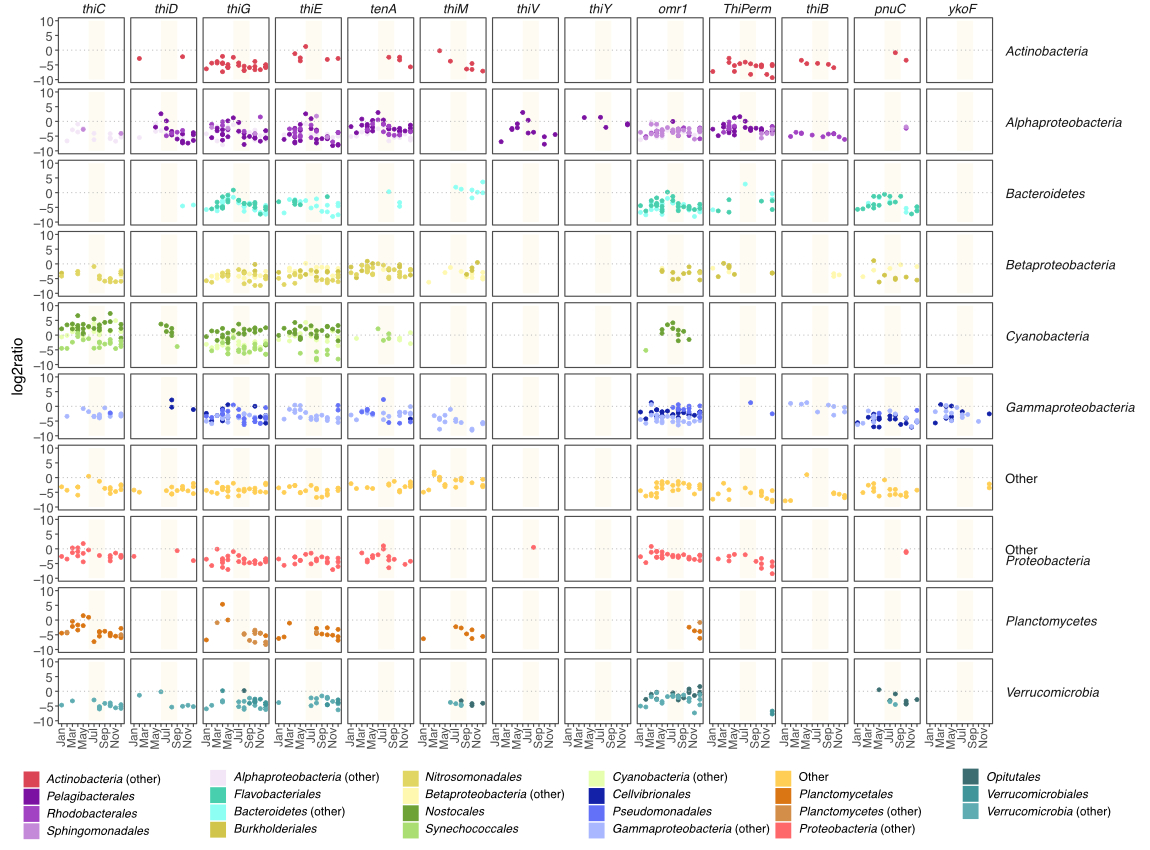


**Supplementary Figure S3.** Plot showing log2 transformed ratios between metatT and metaG relative abundances for each B1 gene, taxon, and time point Positive values indicate higher relative abundance in the metaT, while negative values represent higher relative abundance in the metaG. Yellow shading indicates summer periods.


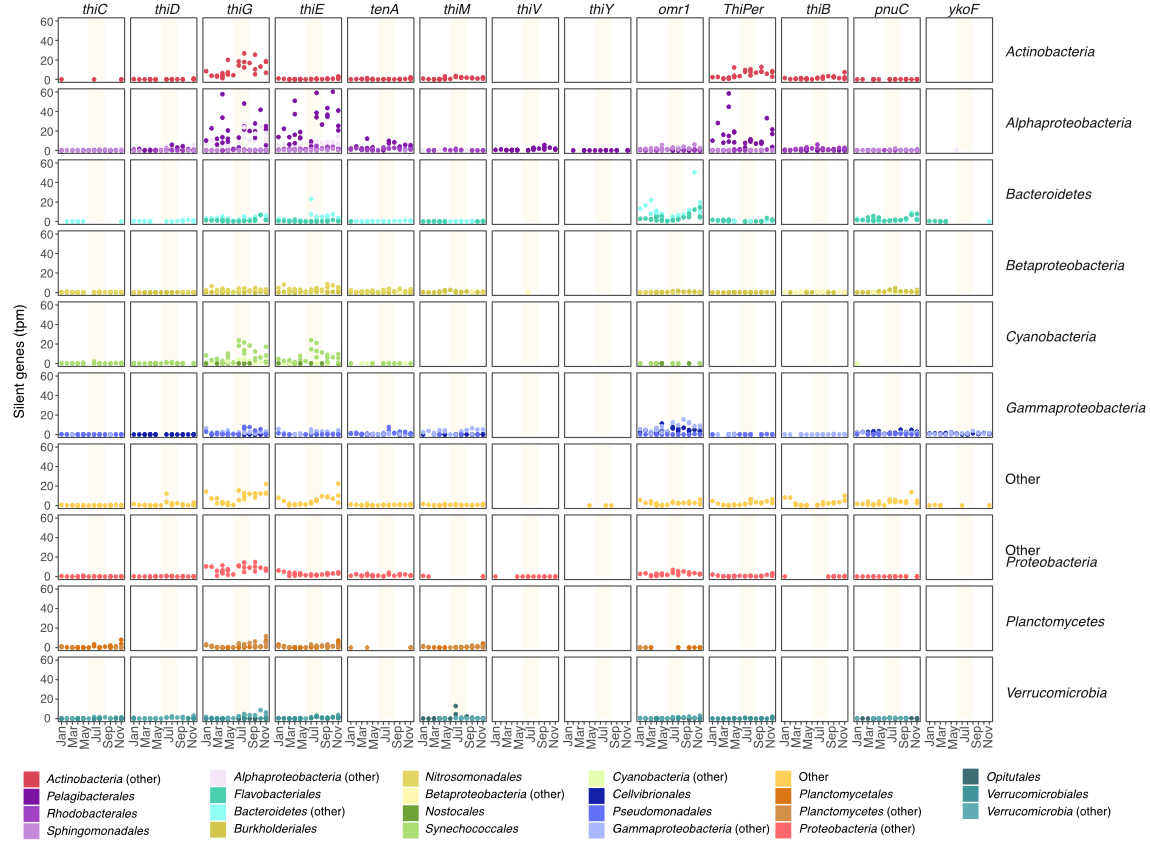


**Supplementary Figure S4.** Abundances of silent genes (genes in the metagenomic dataset and not present in the metaT), for each of main taxa contributing to B1 expression at LMO across the year. Each point represents a sampling. Yellow shading indicates summer periods.
